# Assessing agronomic performance, chocolate spot resistance, and heat tolerance for diverse *Vicia faba* genotypes under varying environmental conditions

**DOI:** 10.1038/s41598-024-59079-3

**Published:** 2024-04-22

**Authors:** Mostafa G. El-Abssi, Hassan A. Awaad, Naglaa Qabil, Elsayed Mansour

**Affiliations:** Department of Crop Science, Faculty of Agriculture, Zagazig University, Zagazig, 44519 Egypt

**Keywords:** Climate change, Yield characters, Chocolate spot, Heat tolerance, Timely sowing, Late sowing, Principal components, Cluster analysis, Heatmap, Natural variation in plants, Plant breeding

## Abstract

Chocolate spot and heat stress devastatingly impact the production of faba bean, particularly under prevailing climatic changes and rising drastic environmental conditions. Hence, the adaptability of faba bean performance is a decisive objective of plant breeders to ensure its sustainable production. The present study aimed to evaluate the agronomic performance and stability of diverse eleven faba bean genotypes for yield characters, chocolate spot, and heat stress in eight different growing environments. The faba bean genotypes were evaluated at two sowing dates in two different locations during two growing seasons. The evaluated eleven faba bean genotypes were sown timely in autumn (25 October) and late sowing in early winter (25 November) in Bilbeis and Elkhatara during 2020 and 2021 growing seasons. The results exhibited substantial differences among the evaluated sowing dates, locations, and faba bean genotypes for all studied characters. The genotypes Sakha-3, Nubaria-3, Nubaria-5, Misr-3, and Wadi-1 were able to produce acceptable yield and quality characters under timely sowing in autumn and late sowing in early winter in all tested environments. Moreover, the genotypes Nubaria-3, Nubaria-4, Nubaria-5, Sakha-4, Giza-3, and Triple White exhibited better resistance to chocolate spot. The assessed faba bean genotypes were evaluated under late sowing to expose the plants to high temperature stress at flowering and throughout the anthesis and seed-filling stages. The genotypes Nubaria-5, Nubaria-3, Nubaria-4, Sakha-3, Sakha-4, Wadi-1, and Misr-3 possessed tolerance to heat stress more than the other genotypes. Different statistical methods were applied to study the stability of assessed genotypes such as joint regression, Additive Main Effect and Multiplicative Interaction (AMMI) analysis, AMMI stability value, Wricke's and Ecovalence values. The estimated stability parameters were consistent in depicting the stability of the assessed faba bean genotypes. The findings revealed that Sakha-1, Misr-3, Nubaria-4, and Nubaria-5 demonstrated stable and desirable performance across all tested environments. The heatmap was employed to classify the assessed faba bean genotypes into different groups based on agronomic performance, chocolate spot resistance and heat stress tolerance. Nubaria-3, Nubaria-4, Nubaria-5, and Misr-3 had the best performance for agronomic performance, chocolate spot resistance, and heat stress tolerance. The obtained results provide evidence of employing promising faba bean genotypes for improving the stability of agronomic performance, chocolate spot resistance, and heat stress tolerance in breeding programs principally under unprecedented climate fluctuations.

## Introduction

Faba bean (*Vicia faba* L.) is an essential pulse crop that has valuable protein for livestock and human nutrition^1,2^. Furthermore, it plays a valuable role in increasing soil fertility through biological nitrogen fixation, particularly in poor soils^3^. Fab bean is one of the few crops that provide valued dietary and simultaneously preserve soil fertility to enhance the agricultural production system^4^. Its global area is around 3 × 10^6^ ha with an annual production of about 7.7 × 10^6^ tons^5^. Egypt is presented in these values by 74 × 10^3^ ha produces 296 × 10^3^ tons. Although faba bean production is decreased annually and the percentage of self-sufficiency is declined^6^. Besides, its production faces great restrictions due to the deleterious impacts of abrupt climate fluctuations and population growth^7,8^. Accordingly, enhancing faba bean production has become an irreplaceable demand to diminish the gap between local consumption and production to enhance global food security﻿.

Biotic and environmental stresses including chocolate spot and high temperature cause devastating influences on faba bean production in particular under the current climate crisis^9,10^. Chocolate spot caused by *Botrytis fabae* is a common fungal infection in different regions worldwide that generates substantial devastation in faba bean growth resulting in considerable production loss^11,12^. Therefore, faba bean breeders focus on identifying resistant genotypes to maintain acceptable productivity and sustain its production^13^. Moreover, it is expected to increase the negative impacts of chocolate spot under current fluctuations of climate variables especially relative humidity, and minimum and maximum temperatures^14^. Besides, the climatic extremes involving high temperatures are expected to increase and destructively impact faba bean productivity^15^. In addition, high temperatures considerably affect physio-biochemical and anatomical parameters in plants, ultimately damaging plant growth and productivity^16^. Substantially, heat stress has a drastic influence on faba bean plants at any growth stage from germination to the reproductive stage^17,18^. However, high temperatures at the reproductive stage are more threatening since they have a greater effect on productivity^9^. Heat stress at critical stages such as flowering, anthesis, and seed filling causes substantial yield reduction in faba bean due to deleterious impacts on pollen germination, growth, elongation, seed setting, and seed filling^19^.

The variability in soil and climate conditions significantly impacts faba bean production, particularly under prevailing climatic changes^20^. This variability introduces a complex challenge for faba bean breeders due to the genotype by environment interaction (G × E), which is pivotal in determining plant performance across diverse environments^21–25^. Consequently, faba bean breeders frequently study the genotype through environmental interaction across different environments to examine the stability of available plant material^26^. Several statistical models e.g., AMMI biplot, regression slope (b_i_), deviation from linear regression (S^2^d_i_), and Wricke’s Ecovalence (WE) are employed to analyze G × E, aiding in the identification of broadly adaptable genotypes or those tailored explicitly to specific environments^27–29^. Considering the genetic basis of the adaptability enhances breeding attempts in developing newly resilient and desired faba bean genotypes under global climate variations^22,30^. Hence, the present study aimed at exploring the adaptability of diverse faba bean genotypes in agronomic performance, chocolate spot resistance, and heat resilience under a Mediterranean environment using different statistical analyses.

## Results

### Analysis of variance

The combined analysis of variance exhibited significant effects for environments (E), genotypes (G) and their interaction (G × E) as presented in Table 1. The studied environments displayed the largest proportion of sum of squares for number of branches per plant and number of pods per plant, seed yield, and aboveground biomass followed by genotypic and interaction effects. Otherwise, the assessed genotypes possessed the largest proportion for 100-seed weight and chocolate spot resistance followed by environmental and interaction effects. While the G × E interaction exhibited the largest proportion of protein content followed by environmental and genotypic effects. Sowing date and tested locations presented the largest proportion of the environmental variation for most studied characters. The genotype by environment interaction (G × E) was significant for all evaluated characters. Moreover, the two-way and three-way interactions among genotype, sowing date, and location were significant for all characters except protein content and chocolate spot resistance.

**Table 1:** Analysis of variance for the studied characters of eleven faba bean genotypes tested in two different locations (Bilbeis and Elkhatara) during two seasons in 2020–21 and 2021–22 under timely sowing in autumn and late sowing in early winter.

Sources of variation	df	NoB/P		NoP/P		100-SW		SY		AgB		Protein		Chocolate	
		MS	SS%	MS	SS%	MS	SS%	MS	SS%	MS	SS%	MS	SS%	MS	SS%
Environment (E)	7	18.122**	52.47	468.5**	48.23	1759**	28.39	19.18**	46.31	143**	47.81	33.78**	29.13	4.67**	12.63
*Season (S)*	*1*	7.168**	2.96	391.5**	5.76	2408**	5.55	3.33**	1.15	103.1**	4.92	23.75**	2.93	15.03**	5.81
*Location (L)*	*1*	16.152**	6.68	1512**	22.25	162.5**	0.37	62.54**	21.58	32.61**	1.56	0.002NS	0.001	0.31NS	0.12
*Sowing date (D)*	*1*	69.341**	28.68	328.1**	4.83	1204**	2.78	28.2**	9.73	711.2**	33.96	4.83NS	0.6	16**	6.18
*S* × *L*	*1*	26.284**	10.87	713.1**	10.49	2815**	6.49	8.24**	2.84	0NS	0	20.9**	2.57	0.004NS	0.001
*S* × *D*	*1*	0.934**	0.39	16.15**	0.24	393.5**	0.91	0.9**	0.31	75.23**	3.59	1.53NS	0.19	0.85NS	0.33
*L* × *D*	*1*	0.07NS	0.03	294.2**	4.33	2962**	6.83	0.6**	0.21	2.59*	0.12	0.003NS	0.001	0.31NS	0.12
*S* × *L* × *D*	*1*	6.906**	2.86	23.58**	0.35	2367**	5.46	30.43**	10.5	76.58**	3.66	185.44**	22.84	0.19NS	0.07
Genotype (G)	10	6.274**	25.95	71.37**	10.5	2255**	51.98	4.68**	16.13	49.33**	23.55	4.83**	5.95	8.24**	31.85
G × E	70	0.498**	14.409	36.29**	37.36	104.6**	16.88	1.41**	33.97	6.48**	21.64	3.72**	32.07	0.79*	21.26
*S* × *G*	*10*	0.268**	1.11	36.32**	5.34	145.3**	3.35	0.81**	2.81	10.39**	4.96	2.91*	3.58	1.53**	5.9
*L* × *G*	*10*	0.67**	2.77	16.86**	2.48	79.32**	1.83	0.76**	2.63	8.49**	4.06	6.25**	7.7	1.4**	5.4
*D* × *G*	*10*	0.383**	1.58	43.96**	6.47	96.88**	2.23	1.08**	3.72	3.52**	1.68	2.58NS	3.18	0.96*	3.72
*S* × *L* × *G*	*10*	0.291**	1.21	41.89**	6.16	146.3**	3.37	2.41**	8.33	5.36**	2.56	3.37*	4.15	0.8NS	3.07
*L* × *D* × *G*	*10*	0.941**	3.89	30.19**	4.44	143.8**	3.32	0.93**	3.22	4.53**	2.16	3.35*	4.13	0.3NS	1.15
*S* × *D* × *G*	*10*	0.66**	2.73	58.71**	8.64	64.2**	1.48	2.05**	7.07	8.8**	4.2	2.63NS	3.24	0.24NS	0.94
*S* × *L* × *D* × *G*	*10*	0.27**	1.12	26.08**	3.84	56.18**	1.3	1.8**	6.2	4.22**	2.02	4.96**	6.1	0.28NS	1.07
Residual	174	0.097	7.0	1.49	3.8	6.81	2.73	0.06	3.47	0.83	6.9	1.53	32.73	0.51	34.11
Total	263	0.92		25.85		164.9		1.10		7.96		3.09		0.98	

*NoB/P* number of branches/plant, *NoP/PN* number of pods/plant, *100-SW* 100- seed weight (g), *SY* seed yield (kg/ha), *AgB* Aboveground biomass (kg/ha), *Protein* Protein content (%), and Chocolate: Chocolate spot resistance. *MS* mean square, *SS***%** percentages of sum of squares.

*P < 0.05.

**P < 0.01.

### Agronomic performance

The assessed genotypes exhibited highly significant differences for all evaluated agronomic attributes under different tested environments. All evaluated genotypes produced higher agronomic characters under timely sowing in autumn compared to late sowing in early winter (Tables 2–4). Number of branches per plant fluctuated from 1.23 to 5.80 (Table 2). The uppermost number of branches per plant was produced by Nubaria-3 followed by Sakha-1 and Giza-3 at E1 while Triple White exhibited the lowest values at E6, E5, and E7 (Table 2). Likewise, Number of pods per plant differed from 10.9 to 35.1 (Table 2). The highest was recorded by Nubaria-5 at E5, Triple-White at E1, E5 and E7, Wadi-1 at E5, and Giza-3 at E5. Otherwise, the minimal values were assigned for Sakha-3 and Misr-3 at E2 and Sakha-1 at E8. The seed index ranged from 45.3 to 104.4 g (Table 3). Nubaria-3, Nubaria-4, Sakha-4, and Wadi-1 at E5 displayed the greatest seed index, followed by Giza-3 at E1 and Nubaria-5 at E5. However, the lowest values were presented by Triple White in all evaluated environments. Seed yield contrasted from 947 to 6550 kg/ha (Table 3). The superior seed yield was produced by Sakha-3 at E5 followed by Wadi-1, Nubaria-3, Giza-3, and Giza-843 at E7. Whereas Triple White displayed the lowest value at E2, E4, and E6. Aboveground biomass altered from 3320 to 15,710 kg/ha (Table 4). The uppermost values were obtained by Nubaria-3 at E5 followed by Nubaria-5, Sakha-3, and Misr-3 at E1 and Sakha-1 at E5. However, the lowest values were given by Triple White and Misr-3 at E2. Protein content varied from 21.35 to 28.27% (Table 4). Nubaria-3, Misr-3, Giza-843 and Sakha-3 at E5 were the greatest values, followed by Nubaria-4 and Wadi-1 at E1. However, the minimum values were recorded by Nubaria-3, Giza-3, and Giza-843 at E2.

**Table 2:** Number of branches and pods per plant of the evaluated faba bean genotypes in 8 environments through two locations (Belbeis and Elkhatara) during two seasons in 2020–2021 and 2021–2022 under timely sowing in autumn and late sowing in early winter. E1-E8 are the tested environments as shown in Table 7.

Genotype	E1	E2	E3	E4	E5	E6	E7	E8	Mean
Number of branches per plant									
Giza-3	5.40	3.53	3.33	3.13	3.53	2.43	3.57	2.60	3.44
Giza-843	4.93	3.63	3.47	3.07	3.03	2.63	4.13	2.53	3.43
Misr-3	4.67	2.90	3.20	3.17	4.73	2.23	4.07	2.70	3.46
Nubaria-3	5.80	3.53	4.07	3.27	3.37	3.33	4.03	3.93	3.92
Nubaria-4	4.40	3.67	3.87	2.73	4.03	3.17	4.37	3.20	3.68
Nubaria-5	4.80	4.40	3.87	3.00	4.33	2.83	4.27	2.53	3.75
Sakha-1	5.43	3.33	3.73	2.57	2.57	2.23	3.43	2.33	3.20
Sakha-3	5.03	3.60	4.00	2.70	4.17	3.17	4.73	2.47	3.73
Sakha-4	5.00	3.40	3.40	2.37	2.63	2.60	3.67	2.73	3.23
Triple white	3.63	1.73	1.80	1.73	1.67	1.23	2.53	1.67	2.00
Wadi-1	4.40	3.27	3.53	3.37	3.13	2.67	4.07	2.80	3.40
Mean	4.86	3.36	3.48	2.83	3.38	2.59	3.90	2.68	
LSD_g_	0.29								
LSD_e_	0.23								
LSD_g×e_	1.08								
Number of pods per plant									
Giza-3	17.73	17.47	15.73	14.67	26.40	23.4	24.40	14.13	19.24
Giza-843	20.80	19.07	14.40	14.27	23.67	22.53	20.40	17.20	19.04
Misr-3	13.20	11.20	14.87	14.87	27.20	27.00	14.80	12.67	16.98
Nubaria-3	16.07	13.47	15.13	13.53	25.87	22.33	16.33	12.73	16.93
Nubaria-4	13.50	13.07	18.53	13.67	18.40	18.40	19.90	12.80	16.03
Nubaria-5	12.87	12.53	15.67	12.40	35.13	19.47	17.33	12.33	17.22
Sakha-1	14.07	13.53	16.27	12.33	23.50	18.20	18.20	11.13	15.90
Sakha-3	12.13	10.93	15.53	15.40	18.73	16.33	15.40	15.27	14.97
Sakha-4	14.20	12.63	22.93	12.80	22.67	17.53	23.73	12.60	17.39
Triple white	27.23	15.53	20.53	14.93	27.07	19.10	28.13	18.40	21.37
Wadi-1	15.47	13.60	17.77	12.80	26.87	18.13	20.60	13.53	17.35
Mean	16.12	13.91	17.03	13.79	25.05	20.22	19.93	13.89	
LSD_g_	1.13								
LSD_e_	0.91								
LSD_g×e_	3.22

**Table 3:** Seed index and seed yield of the assessed faba bean genotypes in 8 environments through two locations (Belbeis and Elkhatara) during two seasons in 2020–2021 and 2021–2022 under timely sowing in autumn and late sowing in early winter.

Genotype	E1	E2	E3	E4	E5	E6	E7	E8	Mean
100-seed weight (g)									
Giza-3	101.40	78.67	79.87	72.73	99.93	85.47	81.77	77.17	84.63
Giza-843	80.67	75.33	92.57	92.56	96.23	86.63	82.83	74.03	85.11
Misr-3	92.33	75.33	82.47	81.37	94.93	93.80	80.40	78.17	84.85
Nubaria-3	91.67	74.67	82.90	79.80	104.37	96.00	79.87	75.13	85.55
Nubaria-4	98.00	75.00	84.73	84.10	104.07	89.37	90.50	87.07	89.10
Nubaria-5	96.00	79.33	97.00	83.60	97.27	93.77	90.27	90.13	90.92
Sakha-1	99.00	85.67	95.67	86.17	95.77	95.10	86.73	86.40	91.31
Sakha-3	99.67	73.00	97.27	86.77	93.03	90.80	83.10	71.93	86.95
Sakha-4	102.00	86.67	88.27	81.93	103.17	91.33	83.70	82.27	89.92
Triple white	64.67	45.33	58.77	58.13	63.70	51.87	55.90	52.53	56.36
Wadi-1	97.67	60.67	96.60	80.27	100.60	83.73	75.03	69.43	83.00
Mean	93.01	73.61	86.92	80.67	95.73	87.08	80.92	76.75	
LSD_g_	2.42								
LSD_e_	1.95								
LSD_g×e_	5.03								
Seed yield (kg/ha)									
Giza-3	3333	1510	2517	2237	4010	2493	4893	2627	2953
Giza-843	2660	1643	2967	1650	3737	2540	4837	2553	2823
Misr-3	3363	1577	3353	2640	4773	3060	3903	3563	3279
Nubaria-3	4117	1857	3767	2727	4020	3980	4967	3163	3575
Nubaria-4	2597	1850	3263	2943	4530	2613	3790	3440	3128
Nubaria-5	2753	1850	4347	3183	4680	3680	4593	3213	3538
Sakha-1	2527	1710	3203	2553	4777	3500	4250	2830	3169
Sakha-3	3097	1553	3463	3180	6550	3473	3433	2993	3468
Sakha-4	3197	1880	4043	2727	4263	3800	2710	2077	3087
Triple white	2160	947	2620	1467	1867	1417	3280	2100	1982
Wadi-1	3320	1853	2797	2417	3550	3227	5410	3080	3207
Mean	3011	1657	3304	2520	4251	3071	4188	2876	
LSD_g_	223								
LSD_e_	179								
LSD_g×e_	432

**Table 4:** Aboveground biomass and protein content of the evaluated faba bean genotypes in 8 environments through two locations (Belbeis and Elkhatara) during two seasons in 2020–2021 and 2021–2022 under timely sowing in autumn and late sowing in early winter.

Genotype	E1	E2	E3	E4	E5	E6	E7	E8	Mean
Aboveground biomass (kg/ha)									
Giza-3	12,380	7720	8957	6580	12,420	12,113	12,367	6793	9916
Giza-843	13,737	7800	8143	6307	12,827	8097	12,987	7937	9729
Misr-3	15,027	5060	11,417	9153	13,720	8087	10,310	9573	10,293
Nubaria-3	13,790	8097	9427	8027	15,710	12,420	9580	9120	10,771
Nubaria-4	14,210	9130	10,133	8280	12,120	12,113	13,400	8857	11,030
Nubaria-5	15,240	10,107	12,440	12,100	14,520	9813	10,807	8060	11,636
Sakha-1	10,713	6157	8593	7713	14,773	7793	9293	8517	9194
Sakha-3	15,060	8063	9523	8277	11,043	10,343	10,660	7423	10,049
Sakha-4	11,337	6673	8933	8267	9420	6770	8590	7683	8459
Triple white	7863	3320	6927	5520	6160	5747	9400	5433	6296
Wadi-1	11,750	7100	9287	8863	12,420	7397	13,777	7737	9791
Mean	12,828	7202	9435	8099	12,285	9154	11,015	7921	
LSD_g_	846								
LSD_e_	680								
LSD_g×e_	1554								
Protein content (%)									
Giza-3	25.90	23.33	24.76	23.11	24.67	24.17	24.42	23.18	24.19
Giza-843	24.87	22.40	25.44	23.66	27.03	25.33	24.43	23.07	24.53
Misr-3	25.13	23.40	25.77	22.64	28.17	22.07	25.88	23.67	24.59
Nubaria-3	25.43	21.77	25.69	24.07	28.27	25.27	25.16	24.96	25.08
Nubaria-4	27.50	25.03	25.88	24.79	26.77	23.83	25.29	21.36	25.06
Nubaria-5	25.63	26.23	26.48	25.42	25.83	25.03	23.89	23.67	25.27
Sakha-1	24.17	23.60	26.21	25.29	25.50	22.33	25.43	21.35	24.23
Sakha-3	25.77	25.50	26.42	24.11	27.07	23.57	26.68	26.16	25.66
Sakha-4	25.50	24.23	25.98	23.10	26.47	25.87	25.95	23.45	25.07
Triple white	25.37	25.10	25.42	23.70	26.60	26.00	24.72	23.27	25.02
Wadi-1	26.77	24.50	24.94	23.44	25.83	23.97	24.45	23.63	24.69
Mean	25.64	24.10	25.73	23.94	26.56	24.31	25.12	23.43	
LSD_g_	1.15								
LSD_e_	0.92								
LSD_g×e_	3.28								

### Chocolate spot resistance and heat tolerance

**Figure 1:**
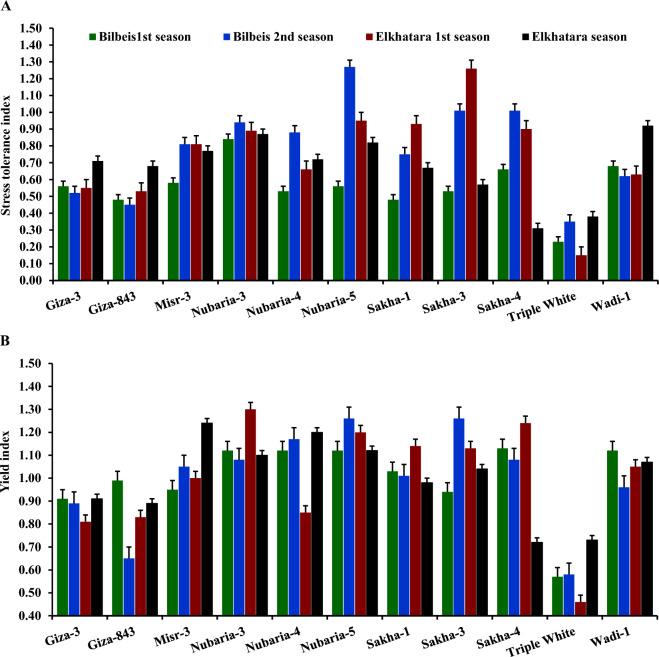
Stress tolerance index and yield index for the assessed eleven faba bean genotypes. The bars on the columns correspond to LSD at p ≤ 0.05.

Highly significant differences were observed among the assessed faba bean genotypes under different environments signifying the presence of genetic divergence (Tables 1 and 5). The results showed that Nubaria-3, Nubaria-4, Nubaria-5, Sakha-4, Giza-3, and Triple White possessed the lowest chocolate spot scores, indicating their resistance (Table 5). Otherwise, Giza-843, Misr-3, Sakha-1, Sakha-3, and Wadi-1 displayed higher scores in the chocolate spot indicating their sensitivity. In general, timely sowing in autumn (E1, E3, E5, and E7) displayed lower chocolate spot compared to late sowing in early winter. Heat stress indices were calculated for the assessed faba bean genotypes across the studied environments (Figs. 1A,B). The tested genotypes possessed highly significant differences in heat stress indices. The genotypes Nubaria-5, Nubaria-3, Nubaria-4, Sakha-3, Sakha-4, Wadi-1, and Misr-3 exhibited statistically significant uppermost stress tolerance index (STI) values under different environments (Fig. 1A). This indicates their tolerance to heat stress. Otherwise, Triple White, Giza-3, and Giza-843 exhibited the lowest STI values suggesting their sensitivity to heat stress. Furthermore, Nubaria-3, Nubaria-4, Nubaria-5, Sakha-3, Misr-3 and Sakha-4 exhibited superior values of yield index (YI), while Triple White, Giza-3, Giza-843 displayed the lowest values of YI (Fig. 1B).

**Table 5:** Chocolate spot resistance for evaluated faba bean genotypes tested in two locations (Belbeis and Elkhatara) during two seasons in 2020–2021 and 2021–2022 timely sowing in autumn and late sowing in early winter.

Genotype	E1	E2	E3	E4	E5	E6	E7	E8	Mean
Giza-3	3.00	4.33	3.00	3.33	3.00	3.67	3.00	3.00	3.29
Giza-843	4.33	4.33	3.67	3.67	4.33	5.00	3.00	3.67	4.00
Misr-3	3.67	4.33	5.00	5.00	4.33	3.67	3.00	3.67	4.08
Nubaria-3	3.67	3.67	3.00	3.00	3.00	3.67	3.00	3.00	3.25
Nubaria-4	3.00	3.00	3.00	3.00	3.00	3.00	3.00	3.00	3.00
Nubaria-5	3.00	3.67	3.00	3.00	3.00	3.67	3.00	3.00	3.17
Sakha-1	3.00	5.00	3.00	4.00	3.67	5.00	3.00	4.33	3.88
Sakha-3	3.67	4.33	3.00	3.67	3.67	4.33	3.00	4.33	3.75
Sakha-4	3.00	3.67	3.00	3.33	3.00	3.00	3.00	3.00	3.13
Triple White	3.67	4.33	3.00	4.33	3.00	3.67	3.00	3.00	3.50
Wadi-1	5.00	6.33	3.00	3.67	5.67	6.33	5.00	5.00	5.00
**Mean**	3.55	4.27	3.24	3.64	3.61	4.09	3.18	3.55	
LSD_g_	0.66								
LSD_e_	0.53								
LSD_g×e_	2.46

### Genotypic classification based on agronomic performance Chocolate spot resistance and heat tolerance.

**Figure 2:**
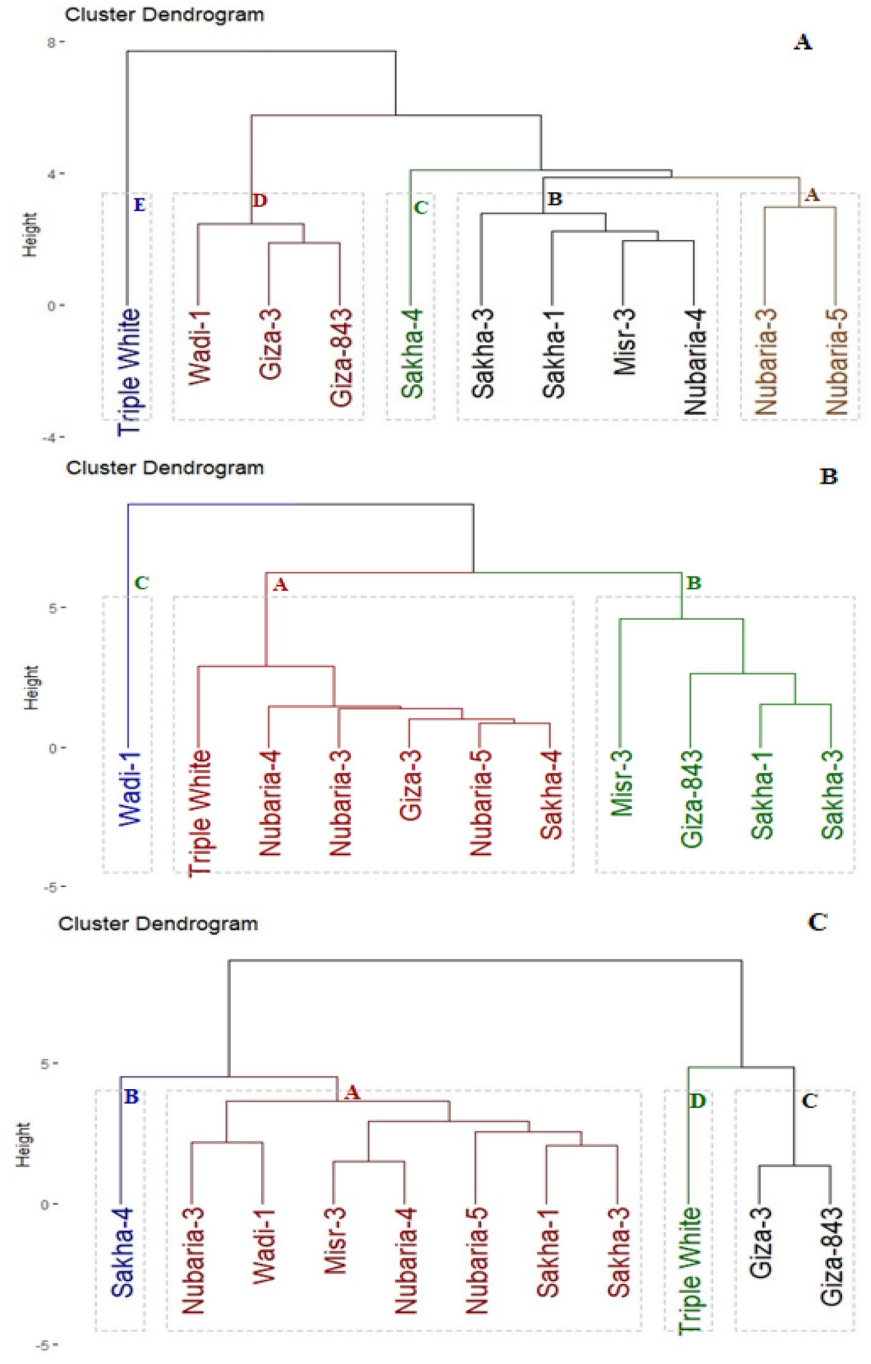
Dendrogram of the phenotypic distances among eleven faba bean genotypes based on yield characters (**A**), chocolate spot resistance (**B**), and heat tolerance indices (**C**) in eight environments.

Yield traits, chocolate spot resistance, and heat tolerance indices were used to categorize the assessed genotypes into different groups based on their performance. The genotypes were clustered utilizing hierarchical clustering into five groups according to yield characters (Fig. 2A). Group A consisted of two genotypes with the greatest yield character values. Groups B, C, and D comprised genotypes that recorded intermediate levels of yield characters. Group E included one genotype with the lowest yield characters. Based on the chocolate spot resistance, the genotypes assessed were differentiated into three clusters (Fig. 2B). Group A contained six genotypes with the highest resistance to chocolate spot. Otherwise, groups B and C included four genotypes and one genotype with intermediate and low resistance to chocolate spot, respectively. Heat tolerance indices (STI and YI) were employed to separate the assessed genotypes into sensitive and tolerant genotypes (Fig. 2C). Four groups were recognized by applying hierarchical clustering. Group A contained seven genotypes that recorded the superior values of tolerance indices STI and YI. Groups B and C comprised one and two genotypes with intermediate values while group D contained one genotype with the minimal values of tolerance indices.

### Seed yield stability

**Figure 3:**
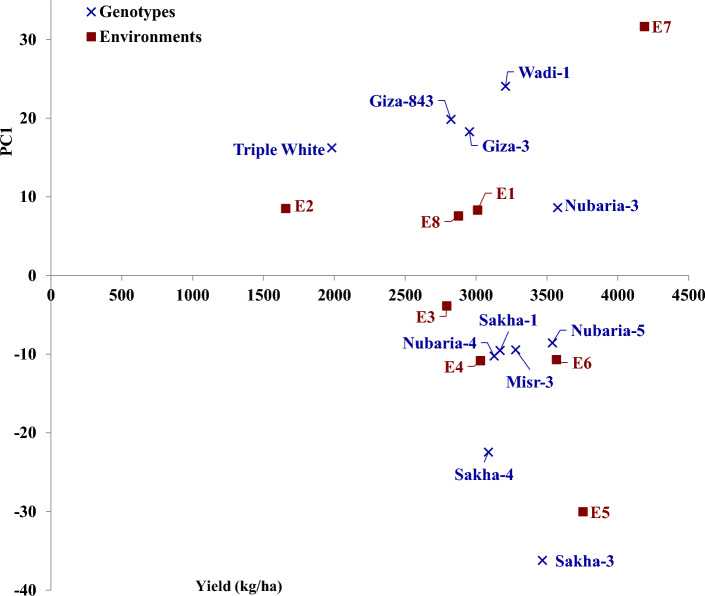
AMMI1 biplot for seed yield against first principal component (PC1) scores of the assessed eleven faba bean genotypes in eight environments (E1-E8) which are shown in Table 7.

**Figure 4:**
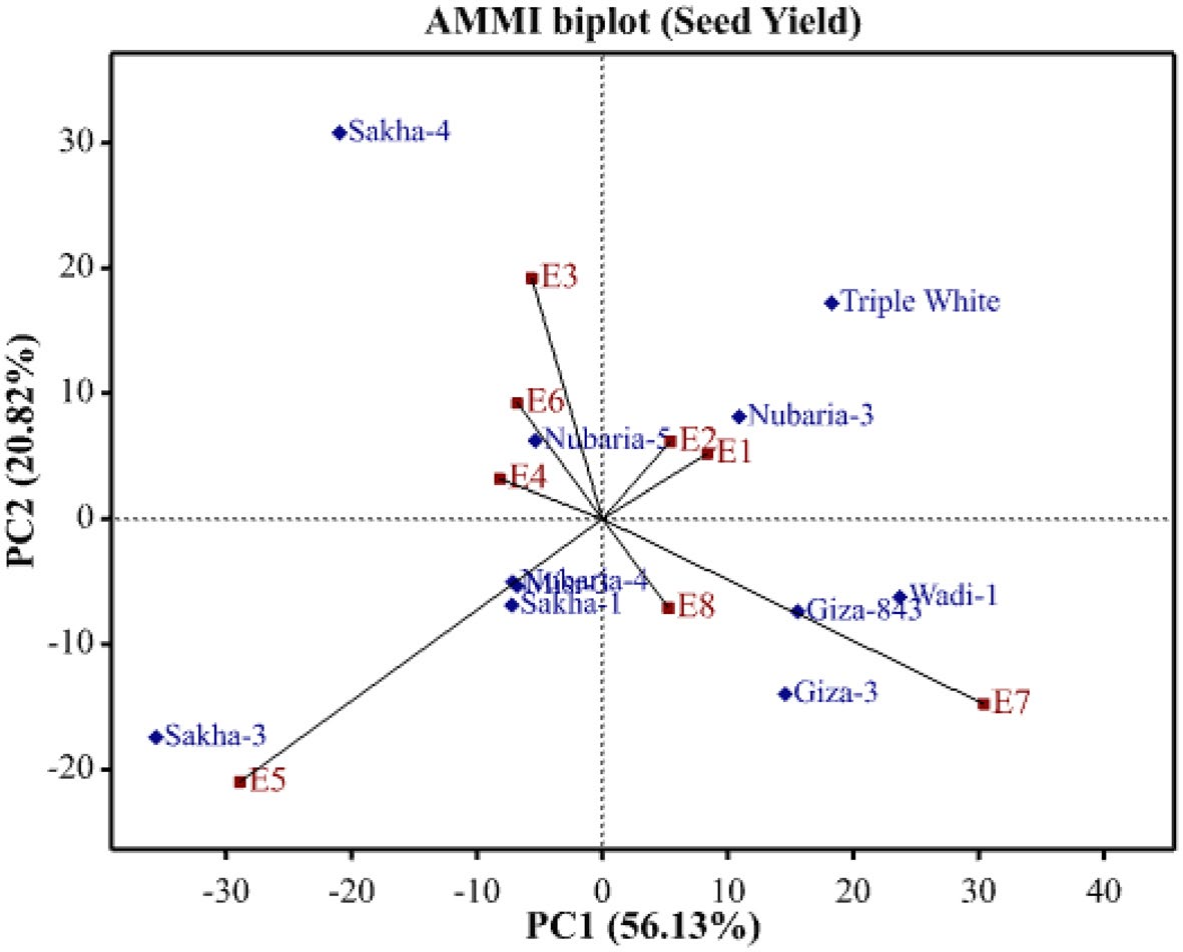
AMMI2 biplot for seed yield of the assessed eleven faba bean genotypes in eight environments (E1-E8) which are shown in Table 7.

Different statistical analyses were applied to study the state of environmental impact on the assessed genotypes. regression slope (bi), deviation from linear regression (S2di), AMMI Stability value (ASV), and Wricke’s Ecovalence (WE) were employed to study genotype stability. Furthermore, the AMMI model was applied due to its effectiveness in graphically recognizing the genotype through environmental interaction. The phenotypic stability revealed that the regression coefficient (bi) for seed yield of assessed faba bean genotypes varied from 0.61 (Triple White) to 1.33 (Sakha-3), exhibiting the genetic variability among the assessed genotypes in their regression response for seed yield (Table 6). The values of regression slope (b_i_) were greater than the unity in genotypes Sakha-3, Giza-3, Giza-843, and Sakha-1, implying their suitability for favorable environments. On the other hand, the regression values (b_i_) were less than unity in Sakha-4, Triple White, and Nubaria-4 suggesting their suitability for unfavorable environments. Whereas the genotypes Nubaria-3, Misr-3, Nubaria-5, Wadi-1 and Sakha-1, displayed b_i_ values approached near the unity indicating their suitability to be grown under a wide range of environments. Moreover, Sakha-1, Misr-3, Nubaria-4, and Nubaria-5 displayed the lowest deviations from regression (S^2^d_i_) for seed yield. Nubaria-5, Misr-3, Nubaria-4, and Sakha-1 displayed the lowest ASV which is the most desired. On the contrary, the other genotypes possessed higher values of ASV and hence more responsive. Sakha-1, Misr-3, Nubaria-3, Nubaria-4, and Nubaria-5 exhibited Wricke’s ecovalence (WE) minimum values. The assessed genotypes displayed diverse PCA scores and hence diverse G × E performance. AMMI1 biplot for seed yield against scores of first principal component (PC1) was performed to represent the effect of genotypes and environments and stability (Fig. 3). The genotypes closer to zero on the PC1 axis indicated a minor contribution to G × E interaction than those further away. The genotypes Nubaria-3, Nubaria-5, Nubaria-4, Sakha-1, and Misr-3 showed high yields and good stability, while Triple White had low seed yield and Sakha-3 was unstable. Besides, the AMMI2 biplot for the first two principal components (PC1 and PC2) was utilized to present the genotype by environment interaction (Fig. 4). The genotypes Nubaria-5, Nubaria-4, and Sakha-1 exhibited minimum PCAs values and were close to the origin of the AMMI biplot. Thus, these genotypes were more stable due to lower G × E interaction (Fig. 4). Otherwise, the genotypes Triple White, Sakha-3, and Giza-3 were situated far from the origin and hence had higher G × E interaction. The studied environments impacted different magnitudes in seed yield deviation. The environments E5 and E7 were the most separating environments, proved substantial contributions to G × E, and were situated away from the origin (Fig. 4).

**Table 6:** Stability parameters of eleven faba bean genotypes evaluated in 8 environments containing two locations (Belbeis and Elkhatara) during two seasons (2020–2021 and 2021–2022) and two sowing dates (timely sowing in autumn and late sowing in early winter).

Genotype	b_i_	S^2^d	ASV	Wricke's ecovalence
Giza-3	1.16	0.23	1.35	1.54
Giza-843	1.13	0.26	1.38	1.41
Misr-3	1.03	0.11	0.62	0.74
Nubaria-3	1.01	0.31	0.99	1.18
Nubaria-4	0.86	0.16	0.65	1.14
Nubaria-5	1.07	0.20	0.51	1.00
Sakha-1	1.12	0.10	0.67	0.69
Sakha-3	1.33	0.68	3.16	5.46
Sakha-4	0.64	0.49	2.08	4.03
Triple white	0.61	0.27	1.69	2.49
Wadi-1	1.01	0.35	2.08	2.39

### Interrelationships among assessed genotypes and studied characters

**Figure 5:**
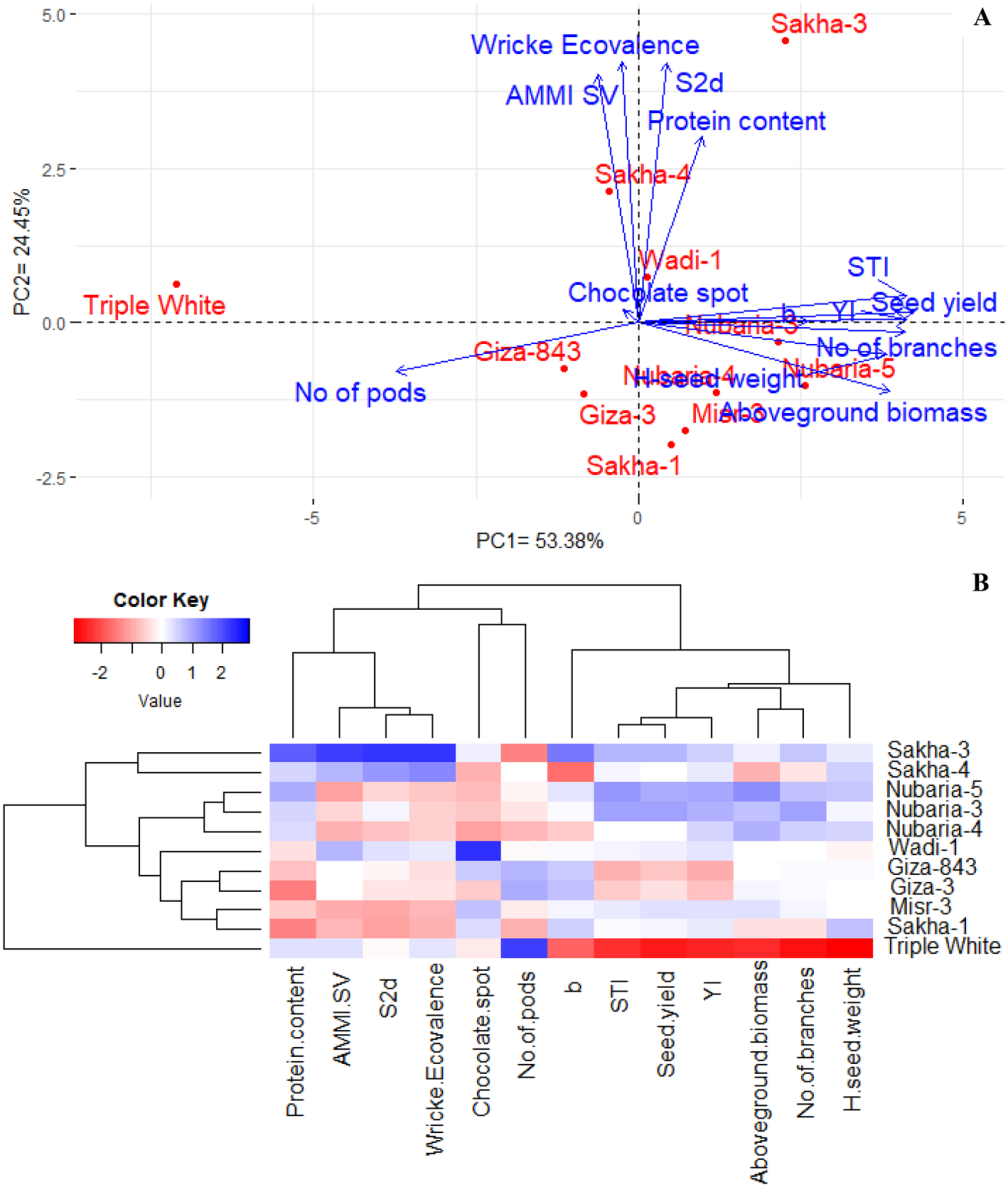
PCA biplot (**A**) and Heatmap (**B**) for the evaluated faba bean genotypes based on agronomic performance, quality traits, rust and chocolate spot resistance.

Principal component analysis was performed to explore the relationship among the assessed faba bean genotypes and studied characters (Fig. 5A). The first two PCs explained 77.83% of the variability. PC1 exhibited 53.38% of the variation and was related to agronomic performance of the evaluated genotypes. PC1 divided the genotypes based on their agronomic performance. High yielding genotypes were located on the positive side, but the lowest performance one was situated on the extremely negative side. The genotypes Nubaria-3, Nubaria-4, Nubaria-5, and Misr-3 were located on the positive side of PC1 and associated with agronomic characters, chocolate spot resistance, and heat tolerance indices. All agronomic characters were positively correlated with these genotypes except for number of pods/plant. This was due to the highest number of pods being assigned for the lowest genotype in agronomic performance, Triple-White. PC2 exhibited 24.45% of the variation and appeared to correspond with the stability performance of tested genotypes. The genotypes were dissimilar, with diverse multidimensional spaces and plots of different distances from bottom to top. The genotypes with low deviation from linear regression (S2di), AMMI Stability value (ASV) and Wricke’s Ecovalence (WE) indicating low genotype by environment interaction were located on the opposite side to these parameters (Sakha-1, Misr-3, and Giza-3). Otherwise, genotypes with high values and high genotypes by environment interaction were situated close to these genotypes (Sakha-3 and Sakha4). Moreover, the heatmap based on the studied characters separated the assessed faba bean genotypes into different clusters (Fig. 5B). The genotypes Nubaria-3, Nubaria-4, Nubaria-5, and Misr-3 displayed superior values for agronomic and quality characters as well as chocolate spot resistance and heat tolerance indices. Besides, these genotypes exhibited the lowest and desired values for stability parameters.

## Discussion

Studying genotypes through environmental interaction assists in translating the results of field trials into beneficial information for plant breeders, particularly under current climate variability. It reflects complex phenotypic responses of different combinations of genotypes, locations, years, agricultural management, and environmental factors and their impact on crop growth, productivity, and seed quality^31,32^. The present study was proposed to assess the adaptability in terms of agronomic performance, seed quality, chocolate spot resistance, and heat tolerance of diverse faba bean genotypes to timely sowing in autumn and late sowing in early winter under two different pedoclimatic locations during two growing seasons. The combinations of different locations, growing seasons, and sowing dates resulted in eight different growing environments with different environmental conditions. The agronomic and quality genes of assessed genotypes were highly impacted by the tested combinations of sowing dates, locations, and growing seasons. This was due to the environmental conditions' variation and their interaction with evaluated genotypes. Appropriately, the environmental effect demonstrated the largest proportion of sum of squares for most of the studied agronomic characters. Indeed, sowing dates and locations exhibited the largest proportion of the environmental variation for studied characters. Several previously published reports deduced that environmental factors are the main considerations affecting faba bean seed yield^32–34^.

In this context, Sellami et al.^31^ examined the effects of sowing date on yield and yield components of *Vicia faba* genotypes, highlighting how these factors contribute significantly to the variation in seed yield and quality traits under Mediterranean field conditions​​. Besides, Greveniotis et al.^29^ extensively evaluated faba beans across multiple locations to assess the influence of G × EI on quantitative and qualitative traits. By employing AMMI biplot model alongside stability indices, this study elucidated the relationship between various ecosystems and faba bean genotypes. It aimed to identify genotypes that offer stable performance across different environments, which is critical for optimizing faba bean production under varying climatic conditions​. Moreover, Papastylianou et al.^22^ highlighted the significant impact of environmental factors on biomass yield, seed yield, plant height, and earliness traits, demonstrating the critical role of G × EI in faba bean breeding and cultivation. This work is relevant for understanding the complexities of adapting faba bean cultivation to varying environmental conditions, thereby informing breeding programs to enhance resilience and productivity​.

Highly significant differences were detected among the assessed genotypes in all evaluated characters, implying genetic variability. All evaluated genotypes produced higher agronomic characters under timely sowing in autumn compared to late sowing in early winter. This was maybe due to the appropriateness of the environmental conditions for the development and growth of faba beans at timely sowing in autumn compared to late sowing in early winter^23,35^. The sowing date regulates available climate variables for the grown plans as precipitation amount, intercepted solar radiation, and exposed temperatures at different plant stages^36,37^. The plants grown in timely sowing in autumn received more precipitation during an extended growing period and were subjected to appropriate solar radiation and lower temperatures compared to late sowing in early winter. Moreover, the reduction of agronomic performance in late sowing could be due to the short maturity period and duration of seed filling, poor plant growth, and reduced seed components. Furthermore, the grown plants in late sowing were exposed to high temperatures at critical stages such as flowering, anthesis, and seed filling which causes adverse impacts on pollen germination, growth, and elongation as well as seed setting and seed filling. In this respect, López-Bellido, et al.^38^; Confalone et al.^37^ and Sellami et al.^31^ elucidated that yield components greatly depend on temperature, particularly during the reproductive stage. The detected substantial G × E interaction for all considered characters revealed that faba bean genotypes contrasted significantly in their response to the environmental variations. In all tested environments, the genotypes Sakha-3, Nubaria-3, Nubaria-5, Misr-3, and Wadi-1 recorded acceptable agronomic and quality characters under timely sowing in autumn and late sowing in early winter.

The assessed genotypes exhibited a wide range of chocolate spot resistance. The genotypes were clustered into different groups based on their resistance to chocolate spot. The results revealed that Nubaria-3, Nubaria-4, Nubaria-5, Sakha-4, Giza-3, and Triple White provided better resistance under all studied environments compared to the remaining genotypes. Otherwise, Giza-843, Misr-3, Sakha-1, Sakha-3, and Wadi-1 had lower levels of resistance and were susceptible to chocolate spot infection. Similarly, Bouhassan et al.^11^; Beyene et al.^13^; Tekalign, et al.^39^; Waly, et al.^40^, and Heiba, et al.^41^ depicted highly significant variations in chocolate spot resistance evaluated in faba bean genotypes. Sowing date considerably affects available climate variables that significantly impact chocolate spot resistance. In general, late sowing recorded higher chocolate spot infection compared to early sowing by providing favorable relative humidity and temperature that are suitable for infection^42^.

Heat stress at critical stages such as flowering and throughout anthesis causes considerable yield reduction due to devastating effects on pollen fertility and seed filling^43,44^. The agronomic performance of faba bean is adversely impacted across altered sowing dates due to heat stress at these critical stages^17,23^. Hence, adapted, and high-yielding genotypes should be assessed for their stability in different sowing dates at different locations in particular under current global warming. Heat tolerant genotypes diminish the deleterious impacts of heat stress at critical stages of flowering and anthesis stages and maintain acceptable crop productivity and quality^18,45^. The faba bean genotypes were evaluated under late sowing to expose the plants to high-temperature stress at flowering and throughout the anthesis and seed-filling stages. Two tolerance indices were applied to distinguish the resilient genotypes and sensitive ones. The results exhibited that Nubaria-5, Nubaria-3, Nubaria-4, Sakha-3, Sakha-4, Wadi-1, and Misr-3 possessed heat tolerance more than the other genotypes. On the other hand, Triple White, Giza-3, and Giza-843 recorded minimum values of STI and YI indicating their sensitivity to high temperatures. Likewise, Lavania et al.^9^,Manning et al.^23^,Sallam, et al.^46^ disclosed substantial differences among faba bean genotypes were assessed under altered sowing dates conditions.

Studying seed yield stability across diverse environments is fundamental for improving faba bean production. Different statistical analyses were applied to study the stability of assessed genotypes such as joint regression, AMMI stability value Wricke's Ecovalence values and AMMI analysis. The employed stability parameters were relatively analogous in describing the stability of the assessed faba bean genotypes. Sakha-1, Misr-3, Nubaria-4, and Nubaria-5 had stable and desirable performance across all tested environments. These genotypes could be exploited in faba bean breeding programs to improve seed yield stability, mainly under prevailing climatic changes and rising drastic environmental conditions^32,47^.

## Materials and methods

### Plant materials and experimental sites

Eleven faba bean (*Vicia faba* L.) genotypes were evaluated in the present study. The genotypes studied with a wide range of diverse genetic materials for agronomic performance were selected for the study. The chosen genotypes represent a mix of commercially adapted varieties and exotic genotypes with diverse genetic backgrounds, as illustrated in Table S1. The genotypes used in this study were obtained from the Legumes Research Department, Agricultural Research Center, Egypt. These genotypes complied with national, international, and institutional legislation and guidelines. The genotypes were evaluated at two varied locations in Egypt; Bilbeis, and Elkhatara during two growing seasons (2020–21 and 2021–22). The trials were sown timely (25 October) and lately (25 November) across both locations and seasons. The evaluated genotypes were sown 30 days after timely sowing to expose the plants to a suitable environment for chocolate spot development, and high temperature during flowering and seed-filling periods. Accordingly, eight field trials were performed at two locations during two seasons at two sowing dates as presented in Table 7. Meteorological data of the tested locations proved by the Egyptian Meteorological Authority are displayed in Table S2. The soil of Bilbeis locations is sandy loam throughout the profile (77.3% sand, 10.7% silt, and 12.1% clay), but Elkhatara is sandy (87.9% sand, 1.6% silt, and 10.6% clay) as presented in Table S3. The experimental design in the eight environments was applied in three replicates using a randomized complete block design. The seeds of the assessed genotypes were sown in plots containing six 4-m long rows with 0.70-m between rows and 0.15 m space between plants. Each hill was planted with four seeds and thinned to two seedlings after full emergence. Phosphorus fertilizer was applied before sowing at a rate of 75 kg P_2_O_5_/ha in the form of calcium superphosphate (15.5% P_2_O_5_). Potassium was added at a rate of 115 kg K_2_O/ha in two equal doses every two weeks after sowing in the form of potassium sulfate (48% K_2_O). Nitrogen was added once at sowing as a basal dose at a rate of 45 kg N/ha in the form of ammonium sulfate (21% N).

**Table 7:** Description of performed field traits.

Code	Location	Latitude	Longitude	Sowing date
E1	Bilbeis	30.35	31.48	In autumn 1st season (25 October 2020)
E2	Bilbeis	30.35	31.48	In early winter 1st season (25 November 2020)
E3	Bilbeis	30.35	31.48	In autumn 2nd season (25 October 2021)
E4	Bilbeis	30.35	31.48	In early winter 2nd season (25 November 2021)
E5	Elkhatara	30.60	31.77	In autumn 1st season (25 October 2020)
E6	Elkhatara	30.60	31.77	In early winter 1st season (25 November 2020)
E7	Elkhatara	30.60	31.77	In autumn 2nd season (25 October 2021)
E8	Elkhatara	30.60	31.77	In early winter 2nd season (25 November 2021)

### Recorded characters

The highly susceptible genotype for chocolate spot (Giza-40) was cultivated regularly after every three genotypes. Also, the field trials were surrounded by a belt of Giza 40 to act as a spreader. The disease severity of chocolate spot resistance was recorded two times at 85 and 115 days after sowing using the scale (1–9) of Bernier, et al.^48^ as presented in Table 8. At physiological maturity when more than 85% of the pods and stems turned black and lost their green pigmentation at about 150 days after sowing yield attributes were recorded. Ten plants were randomly collected from each plot to determine number of branches per plant and number of pods per plant. A hundred-seed weight was determined, and the weight of 100-seeds was tested from each plot. Seed yield and aboveground biomass were measured from harvested four middle rows and converted to tons/ha. The assessed genotypes were assessed for tolerance to high temperature under late sowing as exposed to heat stress during the flowering, anthesis, and seed filling compared to timely sowing. Heat stress tolerance indices were determined as follows: stress tolerance index (STI) = (Ys × Yp)/(Ῡp)^2^ following Fernandez^49^ and Yield index (YI) = (Ys/YP) as outlined by Gavuzzi, et al.^50^. Where Yp is seed yield under normal conditions, Ys is seed yield under stress, Ῡp is the average seed yield of all evaluated genotypes under normal conditions and Ῡs is the average of seed yield under stress conditions.

**Table 8:** Used scale for chocolate spot disease (*Botrytis fabae*).

Score	Symptoms
1	Highly resistant with very specks or no disease symptoms
3	Resistant with few small discrete lesions
5	Moderate-resistant with some coalesced lesions and some defoliation
7	Susceptible with large coalesced sporulating lesions with 50% defoliation and some dead plant
9	Highly susceptible with extensive lesions on leaves stems and pods, severe defoliation, heavy sporulation, stem girdling blackening, and death of more than 80% of plants

### Statistical analysis

A combined analysis of variance over tested environments was performed to study the magnitude effects of the studied environments, assessed genotypes, and their interaction^51^. The regression coefficient (b_i_) and deviation mean square from linear regression (S^2^_d_) were calculated according to Eberhart and Russell^52^. Wricke’s Ecovalence was estimated according to Wricke^53^, AMMI analysis^54^, and AMMI’s stability value (ASV) as disclosed by Purchase^55^. The analyses were performed using the GenStat (version 19).

### Ethical approval

This article does not contain any studies with human participants or animal performed by any of the authors.

## Conclusions

The assessed faba bean genotypes exhibited substantial variations in yield characters, chocolate spot resistance, and heat tolerance implying the presence of genetic variability in the evaluated plant materials. The genotypes Sakha-3, Nubaria-3, Nubaria-5, Misr-3, and Wadi-1 displayed adequate agronomic and quality characters under timely sowing in autumn and late sowing in early winter in all tested environments. Triple White displayed the lowest agronomic performance in all tested environments except for number of pods per plant. The genotypes Nubaria-3, Nubaria-4, Nubaria-5, Sakha-4, Giza-3, and Triple White had better chocolate spot resistance. Otherwise, Giza-843, Misr-3, Sakha-1, Sakha-3 and Wadi-1 displayed low levels of chocolate spot resistance. Nubaria-5, Nubaria-3, Nubaria-4, Sakha-3, Sakha-4, Wadi-1 and Misr-3 were determined as heat tolerant compared to the other genotypes. On the other hand, Triple White, Giza-3, and Giza-843 were sensitive to heat stress. The determined promising faba bean genotypes will be exploited in the faba bean breeding program to enhance its stability and productivity principally under prevailing climatic changes and rising drastic environmental conditions.

### Supplementary Information

Supplementary Tables.

## Data Availability

The data presented in this study are available upon request from the corresponding author.
