# Supplementary material for: Assessing agronomic performance, chocolate spot resistance, and heat tolerance for diverse Vicia faba genotypes under varying environmental conditions
Source: Sci Rep. 2024 Apr 22;14:9224. doi: 10.1038/s41598-024-59079-3 (PMC11035625; doi:10.1038/s41598-024-59079-3)
Supplement: Supplementary file 1 — Supplementary Tables. [file 41598_2024_59079_MOESM1_ESM.docx]

Table S1. Pedigree and origin of assessed faba bean genotypes

| **Code** | **Genotypes** | **Pedigree** | **Origin** |
| --- | --- | --- | --- |
| G1 | Giza-3 | Giza 1×Dutch 29 | Egypt |
| G2 | Giza-843 | 561/2076/85 Sakha x461/845/83 | Egypt |
| G3 | Misr-3 | 667× (Cairo 241× Giza 461) | Egypt |
| G4 | Nubaria-3 | Selection in Ahnasiaz | Egypt |
| G5 | Nubaria-4 | Selected from Landrace Hammam-3 | Egypt |
| G6 | Nubaria-5 | Selected from Landrace Hammam-10 | Egypt |
| G7 | Sakha-1 | Giza 716×620/283/85 | Egypt |
| G8 | Sakha-3 | Promising line716/402/2001 derived from cross 716 (Giza 461×503/453/83 | Egypt |
| G9 | Sakha-4 | Sakha-1×Giza-3 | Egypt |
| G10 | Triple White | Sudan | Sudan |
| G11 | Wadi-1 | Rena Blanka × Triple white | Egypt |

Table S2. Monthly weather data during the field trial at Belbeis and Elkhatara during two growing seasons in 2020-21 and 2021-22.

| Month | Min (°C) | Max (°C) | RH (%) | Rainfall (mm) | Min (°C) | Max (°C) | RH (%) | Rainfall (mm) |
| --- | --- | --- | --- | --- | --- | --- | --- | --- |
|  | Bilbeis | | | | Elkhatara | | | |
|  | First season (2020-21) | | | | | | | |
| October | 19.80 | 34.53 | 57.91 | 0.60 | 18.74 | 32.93 | 60.89 | 0.46 |
| November | 14.66 | 25.71 | 63.56 | 14.13 | 13.37 | 24.83 | 62.84 | 11.98 |
| December | 11.24 | 23.63 | 61.83 | 1.75 | 10.09 | 22.59 | 60.31 | 0.74 |
| January | 9.27 | 22.30 | 62.65 | 4.24 | 8.16 | 21.17 | 61.95 | 3.57 |
| February | 9.14 | 22.53 | 64.91 | 26.18 | 7.75 | 21.52 | 63.05 | 25.48 |
| March | 9.86 | 23.98 | 63.94 | 62.05 | 8.55 | 23.06 | 62.13 | 51.84 |
| April | 12.01 | 30.72 | 51.84 | 1.06 | 10.82 | 29.57 | 54.29 | 0.38 |
|  | Second season (2021-22) | | | | | | | |
| October | 18.49 | 32.73 | 56.36 | 1.87 | 17.28 | 31.23 | 59.05 | 1.34 |
| November | 15.98 | 28.76 | 64.16 | 30.82 | 14.76 | 27.57 | 61.26 | 27.2 |
| December | 10.01 | 20.25 | 70.05 | 14.74 | 8.89 | 19.35 | 66.59 | 12.5 |
| January | 6.33 | 17.58 | 68.90 | 32.25 | 5.24 | 16.58 | 65.17 | 29.99 |
| February | 7.24 | 20.24 | 68.44 | 14.65 | 6.04 | 19.44 | 65.87 | 10.13 |
| March | 7.67 | 21.81 | 61.44 | 19.59 | 6.36 | 20.78 | 59.32 | 13.49 |
| April | 13.63 | 32.59 | 47.87 | 0.00 | 12.47 | 31.19 | 46.38 | 0.00 |

Table S3. Chemical properties of soil of the experimental sites in the two studied locations (Belbeis and Elkhatara)

| **Soil properties** | **Location** | |
| --- | --- | --- |
|  | **Belbies** | **Elkhatara** |
| **Soil particles distribution** |  |  |
| Sand (%) | 77.25 | 87.90 |
| Silt (%) | 10.65 | 1.55 |
| Clay (%) | 12.10 | 10.55 |
| Organic matter (%) | 0.98 | 0.66 |
| Textural class | Sandy loam | Sandy |
| PH | 7.90 | 7.48 |
| EC (dS/m) | 0.68 | 0.77 |
| **Available nutrient (mg/kg soil)** |  |  |
| Nitrogen | 5.13 | 3.01 |
| Phosphorus | 10.17 | 5.55 |
| Potassium | 67.14 | 37.44 |
| **Soluble cations (mmolc/l)** |  |  |
| Sodium | 10.83 | 10.95 |
| Potassium | 10.10 | 10.10 |
| Calcium | 14.00 | 12.00 |
| Magnesium | 12.00 | 11.50 |
| **Soluble anions (mmolc/l)** |  |  |
| Chlorine | 8.10 | 7.00 |
| Bicarbonate | 10.95 | 10.51 |
| Sulphate | 12.93 | 12.05 |
